# Unsupervised subtyping and methylation landscape of pancreatic ductal adenocarcinoma

**DOI:** 10.1016/j.heliyon.2021.e06000

**Published:** 2021-01-18

**Authors:** Shikha Roy, Amar Pratap Singh, Dinesh Gupta

**Affiliations:** Translational Bioinformatics Group, International Centre for Genetic Engineering and Biotechnology, New Delhi, India

**Keywords:** Bioinformatics, Multi-omics, Unsupervised classification, Subtyping, Integratomics, DNA Methylation, RNA-Seq, Pancreatic ductal adenocarcinoma

## Abstract

Pancreatic Ductal Adenocarcinoma (PDAC) is an aggressive form of pancreatic cancer that typically manifests itself at an advanced stage and does not respond to most treatment modalities. The survival rate of a PDAC patient is less than 5%, with a median survival of just a couple of months. A better understanding of the molecular pathology of PDAC is needed to guide research for the development of better clinical treatment modalities for PDAC patients. Gene expression studies performed to date have identified different subtypes of PDAC with prognostic and clinical relevance. Subtypes identified to date are highly heterogeneous since pancreatic cancer is heterogeneous cancer. Tumor microenvironment and stroma constitute a major chunk of PDAC and contribute to the heterogeneity. Better subtyping methods are need of the hour for better prognosis and classification of PDAC for future personalized treatment. In this work, we have performed an integrated analysis of DNA methylation and gene expression datasets to provide better mechanistic and molecular insights into Pancreatic cancers, especially PDAC. The use of varied and diverse datasets has provided valuable insights into different cancer types and can play an integral role in revealing the complex nature of underlying biological mechanisms. We performed subtyping of TCGA-PAAD gene expression and methylation datasets into different subtypes using state-of-the-art normalization methods and unsupervised clustering methods that reveal latent hidden factors, leading to additional insights for subtyping. Differential expression and differential methylation were performed for each of the subtypes obtained from clustering. Our analysis gave a consensus of five cluster solution with relevant pathways like MAPK, MET. The five subtypes corresponded to the tumor and stromal subtypes. This analysis helps in distinguishing and identifying different subtypes based on enriched putative genes. These results help propose novel experimentally-verifiable PDAC subtyping and demonstrate that using varied data sets and integrated methods can contribute to disease prognostication and precision medicine in PDAC treatment.

## 1 Introduction

Studies have shown that epigenetic processes are often changed during different stages of cancer, including the initial stage and progression of tumor stages. The changes also include a global change in the DNA methylation profiles concerning normal DNA methylation patterns [1]. Broadly, this change in DNA methylation is characterized by overall genome-wide hypomethylation and DNA hyper-methylation of CpG island promoters [2, 3]. Many studies have been conducted using the TCGA dataset on DNA methylation in different cancers, which has provided new insights into these cancers [4, 5]. PDAC accounts for most exocrine pancreatic cancer cases, with variants being less common and, apart from differences in prognosis, being uninformative for management decisions [6]. Adenosquamous carcinoma is an uncommon variant of PDAC and shares the features of adenocarcinoma and squamous cell carcinoma, showing a mixture of glandular and squamous differentiation [7]. Other carcinomas of the exocrine pancreas with acinar differentiation include pancreatoblastomas, acinar cell carcinomas, and carcinomas with mixed histology and are usually identified by staining for trypsin [8]. Five-year survival of PC is less than 5%, with survival just a couple of months [8].

Pancreatic ductal adenocarcinoma (PDAC) is an aggressive disease that represents itself at an advanced stage and does not respond to most of the available treatment options [9, 10]. Studies have shown that PDAC is predicted to become the second leading cause of cancer mortality by 2030 [11]. Various studies have shed light and have helped decipher and characterize the PDAC genetic alterations, provided important insights into the biology of the disease, and laid the foundation for the development of approaches for detection and improved therapies. Initial whole-exome sequencing studies of pancreatic cancer identified several factors, including mutations and changes in somatic copy number alterations (SCNAs) that altered the function of many key oncogenes and tumor suppressor genes, including *KRAS, TP53, SMAD4, and CDKN2.* DNA sequencing of neoplastic cells has demonstrated that most PDACs show complex chromosomal rearrangement patterns, some of which are consistent with PDAC progression [12]. Numerous gene expression studies have identified subtypes of PDAC with prognostic and biological relevance [13, 14, 15, 16]. Genomic analyses have previously revealed heterogeneous landscapes of mutation, copy number variation, structural variation, and gene expression in pancreatic cancer [19]. Better understanding and delineating the molecular pathology of pancreatic ductal adenocarcinoma cancer is an urgent need to achieve advances in clinical treatment for patients. Intra-tumoral heterogeneity makes PDAC a complex disease [17]. Tumor microenvironment, stroma, and immune cell filtrate contribute to the heterogeneity in PDAC. Some have considered Intra-tumor heterogeneity as the Rosetta stone of tumor therapy resistance [18]. Thus, there is a need to identify homogeneous groups from different high throughput datasets, which could be an important step towards better-personalized clinical management of PDAC patients. A plethora of large-scale and valuable experimental data has been generated from extensive experimentations. For example, DNA methylation has been explored in many cancers. It has provided novel and valuable insights into many cancers. Epigenetic mechanisms regulate ontological gene expression networks at different levels, including time and place, giving rise to both normal and disease phenotypes. Recently, multiparametric integrative chromatin immunoprecipitation-sequencing (ChIP-seq) studies on multiple histone modifications, RNA-sequencing (RNA-Seq) and DNA methylation studies have been performed to define the epigenetic landscape of various PDAC subtypes [19]. Integrative analysis of TCGA pancreatic ductal adenocarcinoma, involving different varied and diverse datasets, have revealed a complex molecular landscape of PDAC [20, 21, 22]. Survival analyses based on diverse datasets, including RNA-Seq, DNA methylation, miRNA, long non-coding RNA, representing PDAC patients and normal subjects, have led to the identification of putative gene markers associated with survival and prognosis [23]. In this study, we have performed unsupervised clustering and integrative analysis of PDAC DNA methylation and gene expression data, obtained from The Cancer Genome Atlas (TCGA).

Molecular aberrations and alterations identified in cancers often have multiple synergic interactions as it is a complex disease. Thus, it is important to collect and analyze multiple data types to improve patients' prognosis and response to treatment. A single omics screen cannot fully reveal and decipher the complexity of a biological entity. Therefore, studies involving varied datasets help in a better understanding of the system. Integrating the diverse and rich information from diverse datasets has been an approach to identify latent, hidden factors and putative biomarker identification for cancer and non-communicable studies. Integrative approaches in cancer usually focus on integrating multiple types of omics data such as RNA-Seq, DNA methylation, ChIPseq, etc., rather than using a single omics profile. Varied and diverse data has provided valuable insights into different cancer types and can play an integral role in revealing the underlying complex nature of biological mechanisms in breast cancer, colon cancer, and pancreatic cancer [24, 25, 26]. The earliest example of data integration in omics reported in the literature were studies that involved data analysis from individual omics separately, one by one and the results of these parallel studies were then finally merged [27]. The background behind the integratomics or integrative analysis involving different layers of information is based on emergent property in systems theory. The concept of emergent property has become very popular in the systems biology approach. The emergent properties indicate how some system features are observed only when the system is studied as a whole and not as the sum of its parts [28, 29]. The integrative analysis approach will help in providing better mechanistic and molecular insights into pancreatic ductal adenocarcinoma subtypes. TCGA-PAAD dataset gene expression studies to date include the whole data, which involves PDAC and non-PDAC samples. Our analysis takes this heterogeneity into account and includes only matched PDAC gene expression and DNA methylation samples for better data integration. We have explored the subtyping of PDAC by integrating two different levels of data, using state of the art normalization methods and unsupervised clustering method intNMF, which gives latent factors, thus leading to better subtyping. The current study tries to provide better insight into the understanding of DNA methylation underlying PDAC heterogeneity and identification of epigenetically modified regions in different PDAC subtypes leading to better subtyping, which can serve as potential new markers and therapeutic targets. Our integrated data analysis indicates that gene expression, DNA methylation, and tumor-intrinsic factors, such as the tumor microenvironment and immune cell filtrate, all contribute to the heterogeneous landscape of PDAC and thus, the integrative analysis of these factors lead to better subtyping and a better understanding of the distinct PDAC landscape.

## 2 Methodology

### 2.1 Data mining of RNA-Seq and methylation datasets

**Figure 1:**
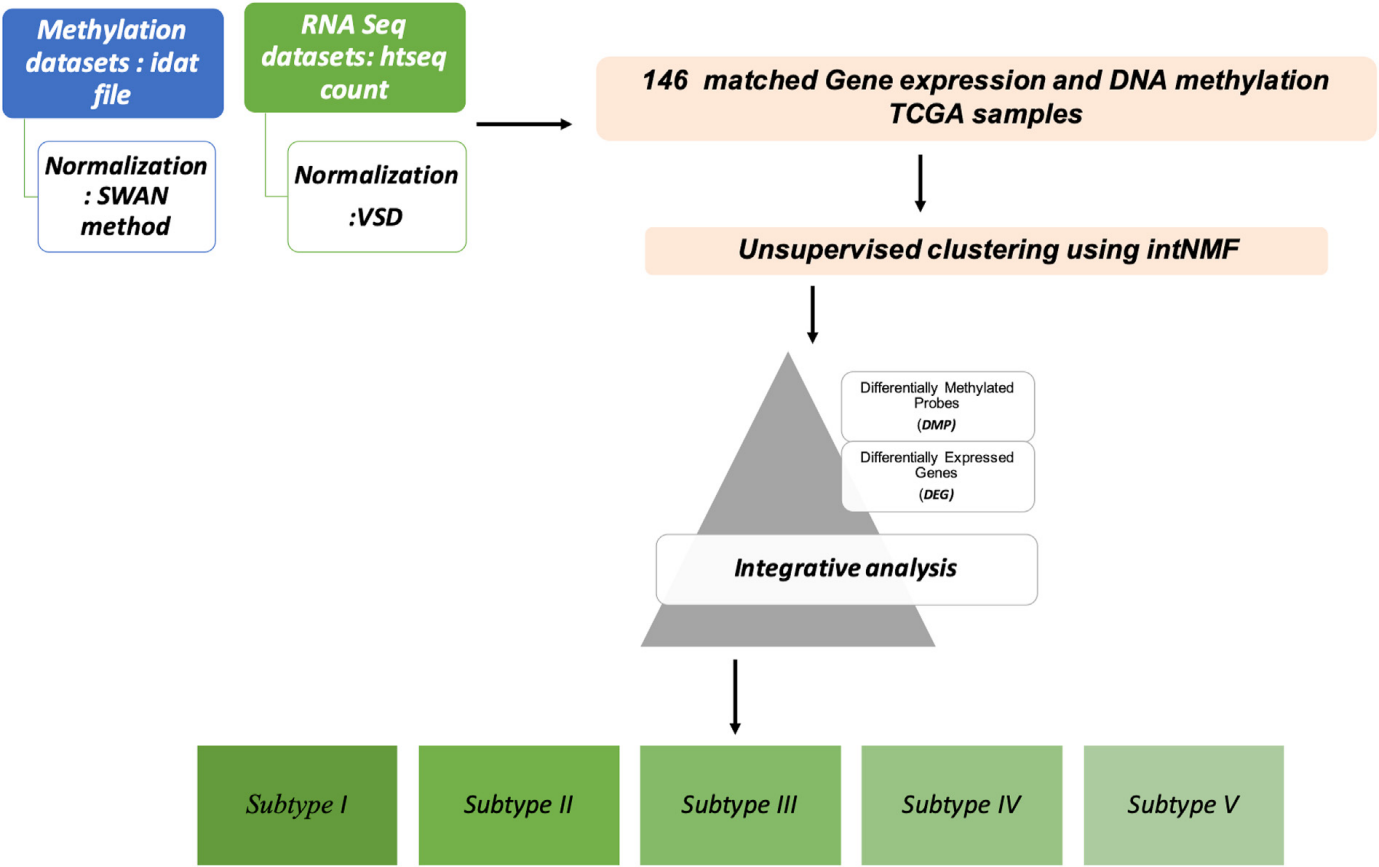
Workflow followed in the study to identify major subtypes of pancreatic cancer by integrative clustering analysis of methylation and genomics datasets.

The study workflow is shown in Figure 1. TCGAbiolinks package was used to obtain pancreatic cancer datasets from TCGA. This package imports and processes molecular profiles from high-throughput experiments such as next-generation sequencing and methylation array and their clinical data for statistical analysis [30]. To date, most of the studies performed on pancreatic cancers have focused on TCGA_PAAD datasets, which is heterogeneous data containing PDAC along with non-PDAC samples. Non-PDAC cancer includes adenosquamous carcinoma, colloid carcinoma, squamous cell carcinoma, and neuroendocrine tumor. Missing and non-matched samples were removed before subtyping and clustering.

### 2.2 Data pre-processing of methylation datasets

ChAMP or chip analysis methylation pipeline is used for the analysis of methylation datasets such as filtering low-quality probes, adjustment for Infinium I and Infinium II probe design, batch effect correction, detects differentially methylated probes (DMPs), differentially methylated regions (DMRs) and detection of copy number aberrations (CAN) [31]. It also can filter SNPS based on user-specific minor allele frequency in one of four populations as defined by the 1000 genomes project [32]. It uses the algorithm “probe lasso” method for DMR hunting that incorporates annotated genomic features and their corresponding local probe densities and methylation [33].

### 2.3 Data normalization of methylation datasets

There are various methods for the normalization of DNA methylation data such as Noob, Subset-quantile within array normalization (SWAN), Beta-Mixture Quantile (BMIQ), and Functional normalization (FN). SWAN performs scaling of Infinium I and Infinium II probes together within a single array to minimize the differences in beta value distribution [34, 35]. BMIQ normalization uses state-membership probabilities under the beta mixture model to reassign quantile to type2 probe based on type1 probe distribution (Supplementary file I, S1) [36]. Noob capitalizes on the Infinium I probe's unique design to perform with-array normalization of methylation datasets (Supplementary file I, S2) [37]. FN is an unsupervised method that improves replication between experiments even in batch effect and uses a control probe to act as surrogates variable for unwanted variation [38].

### 2.4 Data normalization of RNA-Seq datasets

Gene expression datasets of 146 samples were normalized using variance stabilizing normalization (VSN) of the DESeq package. It is used to analyze count datasets generated from high throughput experiments to perform downstream processing such as differential expression analysis [39]. To capture differential signals with high statistical power, its uses a negative binomial generalized linear model where mean and variance are linked to local regression [39]. Data normalization of our datasets was performed using VSN, which uses parametric fit for dispersion using vst function. The vst function calculates variances from fitted dispersion-mean relation and transforms count data into homoscedastic data (https://rdrr.io/bioc/DESeq2/man/varianceStabilizingTransformation.html). Variance stabilized transformed datasets incorporates a correction for size factors or normalization factors.

### 2.5 Identification of subtypes using clustering

Before intNMF, genes with low variability across samples were removed from gene expression datasets and methylation datasets using Mean absolute deviation (MAD). The basic assumption or premise behind it is that genes with high variability contribute more to the clustering process. intNMF package was used for clustering using highly variable genes and methylation probe. This package utilizes a non-negative matrix factorization method to perform unsupervised integrated clustering of high dimensional datasets [40]. The k represents the number of clusters varied across a suitable range and was repeated 20 times to predict the optimum number of clusters based on cluster predictive index (CPI). Parameters used while running intNMF were n.runs = 30, n.fold cross validation = 5, k.range default value = 2 to 8, st.count = 10, maxiter which is maximum number of iteration = 20 and wt = 1 for each data. It generates CPI that is the measure of the stability of clusters obtained. It signifies the correlation between sample distances obtained for the consensus matrix representing the clusters [41]. It also generates a silhouette index value for interpretation and validation of consistency within the data clusters. The technique provides a graphical representation of how well each object has been classified based on the cluster's tightness and separation [42]. The silhouette value can range from −1 to +1, where a high value representing the object is well matched to its cluster and poorly matched neighboring clusters.

### 2.6 Differential methylation analysis of obtained subtypes

ChAMP (The Chip Analysis Methylation Pipeline) package was used to perform differential methylation analysis on five subtypes obtained from intNMF. It is used for the various downstream process in methylation datasets such as normalization, detecting differentially methylated regions and copy number aberrations [31]. Differential methylation analysis is performed by limma that uses a linear model for both categorical variables like two phenotypes, like “tumor”, “metastasis” or “control,” as well as a numeric variable such as age to calculate the p-value for differentially methylated probes. It carries regression analysis to find out covariate-related CpGs for the specified condition. Its output includes some data frames of p-value, t-statistic, difference in mean methylation between two groups (for categorical covariate only), average beta value for sample group, and delta beta value for two comparison groups and annotation for each probe. It also includes the annotation for each probe, the average beta value for the sample group, and the delta beta value for the two groups used in the comparison (https://www.bioconductor.org/packages/release/bioc/vignettes/ChAMP/inst/doc/ChAMP.html). The absolute minimum beta value is 0.2, and for the Benjamini-Hochberg adjustment method, p-value <0.01 is used as a cut-off for DMR analysis.

### 2.7 Differential gene expression analysis of obtained subtypes

Differential gene expression analysis was performed for 5 subtypes obtained from intNMF, using the edgeR [43]. It uses a negative binomial distribution model for gene count and performs differential expression analysis on RNA-Seq expression profiles [44]. It implements a range of statistical methodologies such as empirical Bayes estimation, which generates gene-specific dispersion estimates, ranking genes that behave consistently across the replicates higher than others [45].

### 2.8 Correlation analysis of DMR and DEG for obtained subtypes

The in-depth integrative analysis relies on analyzing multiple datasets such as gene expression, methylation, CNV (copy number variations), etc., to extract and identify major biological insights for disease progression. Correlation between methylation and gene expression was carried out to estimate the extent to which methylation influences gene expression in pancreatic ductal adenocarcinoma cancer [46]. The correlation analysis was carried out using TCGAbiolinks starburst function between differentially methylated CpG sites and differentially expressed genes for each subtype individually. This analysis gives clues regarding the epigenetically regulated genes responsible for heterogeneity in pancreatic ductal adenocarcinoma cancer. Starburst plots generate an exponential curve that captures the non-linear relationship between methylation and gene expression utilizing a gene probe that occurs within 20kb windows from each other [47]. Parameters used in Starburst plots are expression p-value cut-off of 0.05 and methylation p-value cut-off of 0.05. DNA methylation platform used is 450K, and genome of reference used to identify nearest probes is hg38.

### 2.9 Go analysis of obtained subtypes using clusterprofiler to identify gene signatures

Clusterprofiler was used to perform gene ontology analysis on the gene signatures for the predicted subtypes. This package was used for pathway level analysis to obtain a system-level understanding for gene signatures obtained from analysis datasets generated from various platforms such as RNA-Seq, micro-array, etc. [48]. GO was performed on gene signatures returned by the correlation analysis of DMR and DEG to determine major pathways and processes regulated in each of the subtypes using the clusterprofiler.

## 3 Results

### 3.1 Data mining of TCGA_PAAD

**Figure 2:**
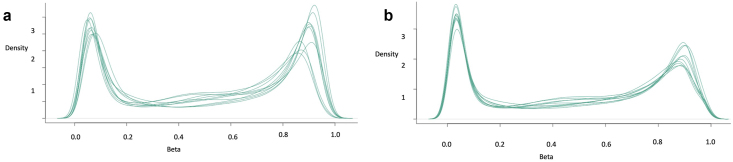
Various normalization methods used for Infinium I and Infinium II probe normalizations. a) SWAN b) Functional normalization. It can be seen from the images that SWAN performs better normalization than functional normalization of Infinium I and Infinium II probe peaks. Hence SWAN normalized datasets were taken further for integrative clustering analysis.

The standard TCGA dataset for pancreatic cancer TCGA-PAAD was downloaded from TCGAbiolink, including 183 cancer and four normal samples. The curation of TCGA_PAAD samples is important for removing biological and clinical biases from non-PDAC samples [49]. PDAC gene expression RNA-Seq datasets and DNA methylation datasets consisted of 153 and 146 samples, respectively. After looking for seven missing samples for DNA methylation data, 146 matched PDAC samples were selected [49]. These 146 PDAC samples consisted of expression profile for DNA methylation as well as gene expression. Gene expression profile and DNA methylation datasets were normalized by various methods before performing clustering and subsequent integration analysis. There are several methods for preprocessing the 450K array data, adjust for probe-type or color bias, subtract background signals, and eliminate systematic errors [50]. DNA methylation datasets were normalized using various methods such as SWAN, BMIQ, Noob, and Functional normalization (FN). SWAN method gave the best result for Infinium I and Infinium II probe normalizations. In SWAN normalization, type I probe density for type I probe is the same as type II probe density, but in funtonorm type I probe density is 4 but type II probe density is 2.55 (Figure 2a,b). SWAN-normalized datasets were taken further for integrative analysis.

### 3.2 Filtering of CpG probes using ChAMP

ChAMP performed initial preprocessing on methylation datasets using idat files. If for a probe, a p-value of detection was above 0.01, it was regarded as a failed probe. The selected probes were filtered with a p-value detection value above 0.01, which resulted in the removal of 54800 probes. It also filtered non-CpG probes resulting in the removal of 1616 probes. It filtered the probes associated with SNP resulting in the removal of 48827 probes. It filtered probes with beadcount less than 3, which lead to the removal of 144 probes in at least 5% of the study samples. On applying Multihit stats, 11 probes were removed. It also filtered probes that are mapping to X and Y chromosomes, which resulted in the removal of 8056 probes.

### 3.3 VSN normalization of RNA-Seq datasets

**Figure 3:**
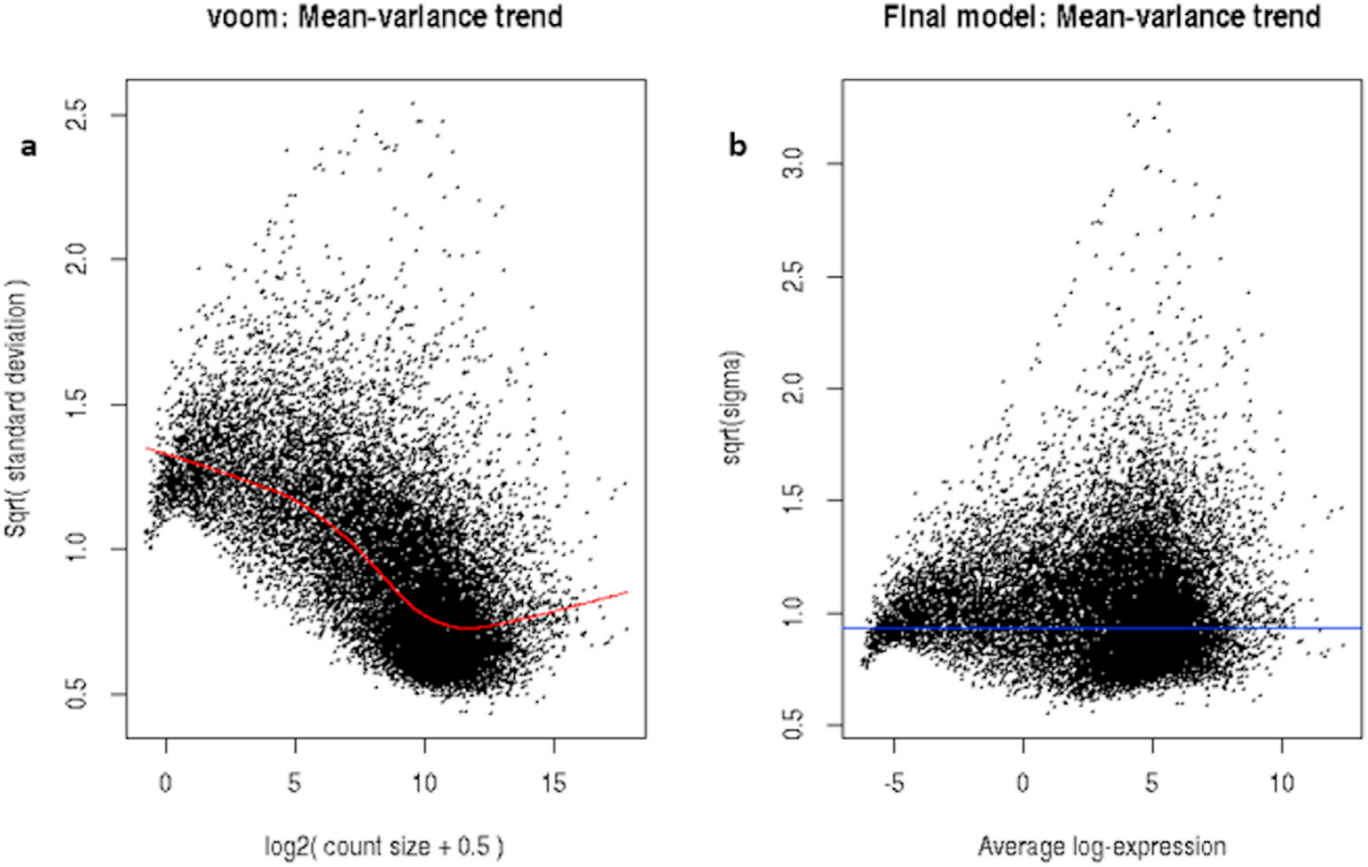
The figure below plots the standard deviation of the transformed data, across samples, against the mean, using the shifted logarithm transformation, the regularized log transformation, and the variance stabilizing transformation. a) The shifted logarithm has elevated standard deviation in the lower count range, and the regularized log to a lesser extent b) In the variance stabilized data the standard deviation is roughly constant along with the whole dynamic range.

VSN was performed on RNA-Seq datasets obtained for TCGA-PAAD patients before performing clustering analysis. Before VSN the expression profile had standard deviation elevated in the lower count range (Figure 3a). After VSN the standard deviation was constant across the expression profile (Figure 3b). Normalization resulted in the transformation of data, which was homoscedastic, with constant variance. It removed the influence of technical variation such that the true biological variation can be discovered.

### 3.4 Identification of subtypes using clustering by intNMF

**Figure 4:**
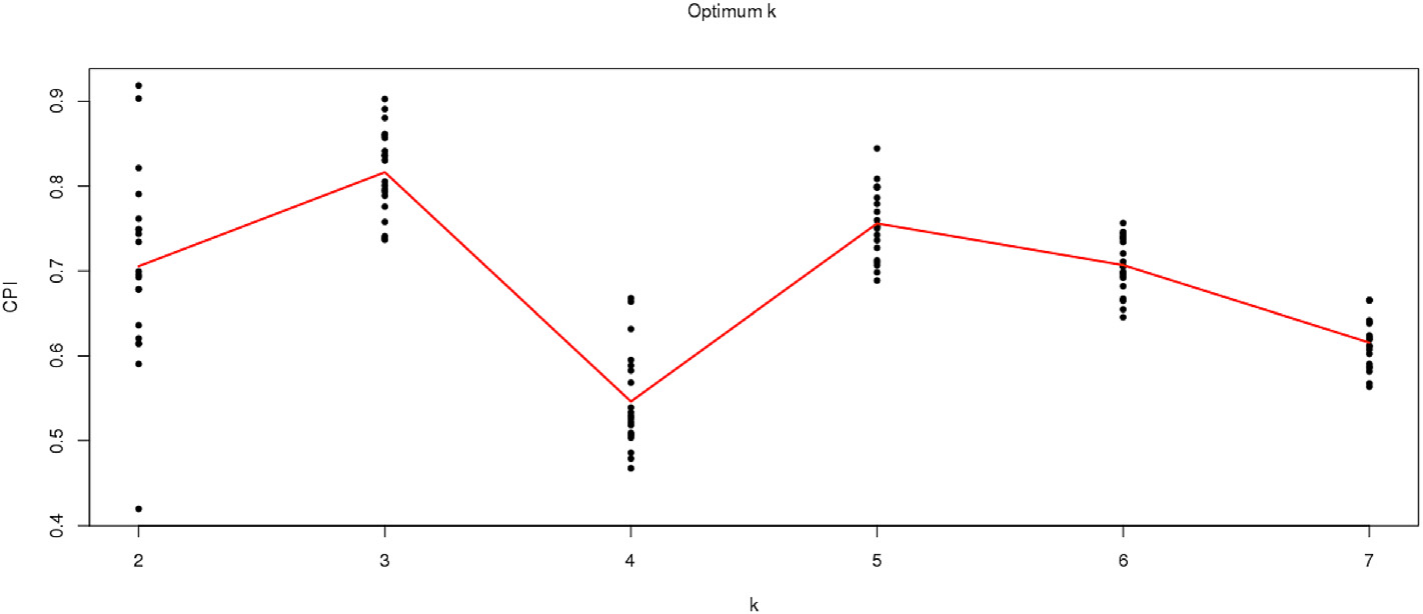
Cophenetic correlation coefficient plot obtained using intNMF. Cophenetic correlation coefficient/Cluster predictive index (CPI) measure of the stability of clusters obtained by intNMF. It signifies the correlation between sample distances obtained for the consensus matrix representing the clusters. We can see from the image that there is high CPI value of 0.76 at five cluster solution.

**Figure 5:**
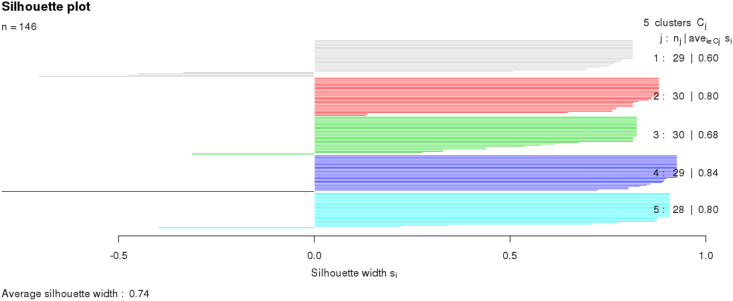
Silhoutte plot obtained for subtypes from clustering analysis by intNMF. It also generates silhouette refers to a method of interpretation and validation of consistency within clusters of data. It also provides a graphical representation of how well each object has been classified based on the tightness and separation of the cluster. Five clusters solution were optimal as they showed the average silhouette width of 0.74.

Before performing clustering, expression profile with low variability across samples was removed for gene expression and methylation datasets. MAD was calculated for each gene and probe, values with less than 0.5 were excluded from our analysis. We have performed MAD (mean absolute deviation) before intNMF as MAD gives highly variable probes. The reason for performing intNMF and no other methods like consensus clustering is that intNMF takes latency and variability into account and thus provides highly significant clusters compared to other methods. It also does not take the data distribution into account, making it highly suitable for analyzing diverse datasets. The PCA analysis has shown no significant variability can be explained by principal components for our multiple datasets (supplementary file I, S3). Subsequently, the top 1000 most variable probes and gene features were used to perform clustering on samples to predict subtypes using intNMF. The intNMF analysis provided optimum cluster solution by k, which was varied from 2 to 10, and the clustering process was repeated 20 times. The value of k corresponding to maximum CPI was chosen as the optimal solution. Our analysis gave an optimum five clusters solution, described in detail below. Since the top 1000 probes have a high CPI of 0.76, these were selected for further analysis (Figure 4). Thus, optimal Five clusters solution was obtained with a high CPI of 0.76 with average silhouette width of 0.74 (Figure 5).

### 3.5 Differential methylation analysis to identify DMPs

**Figure 6:**
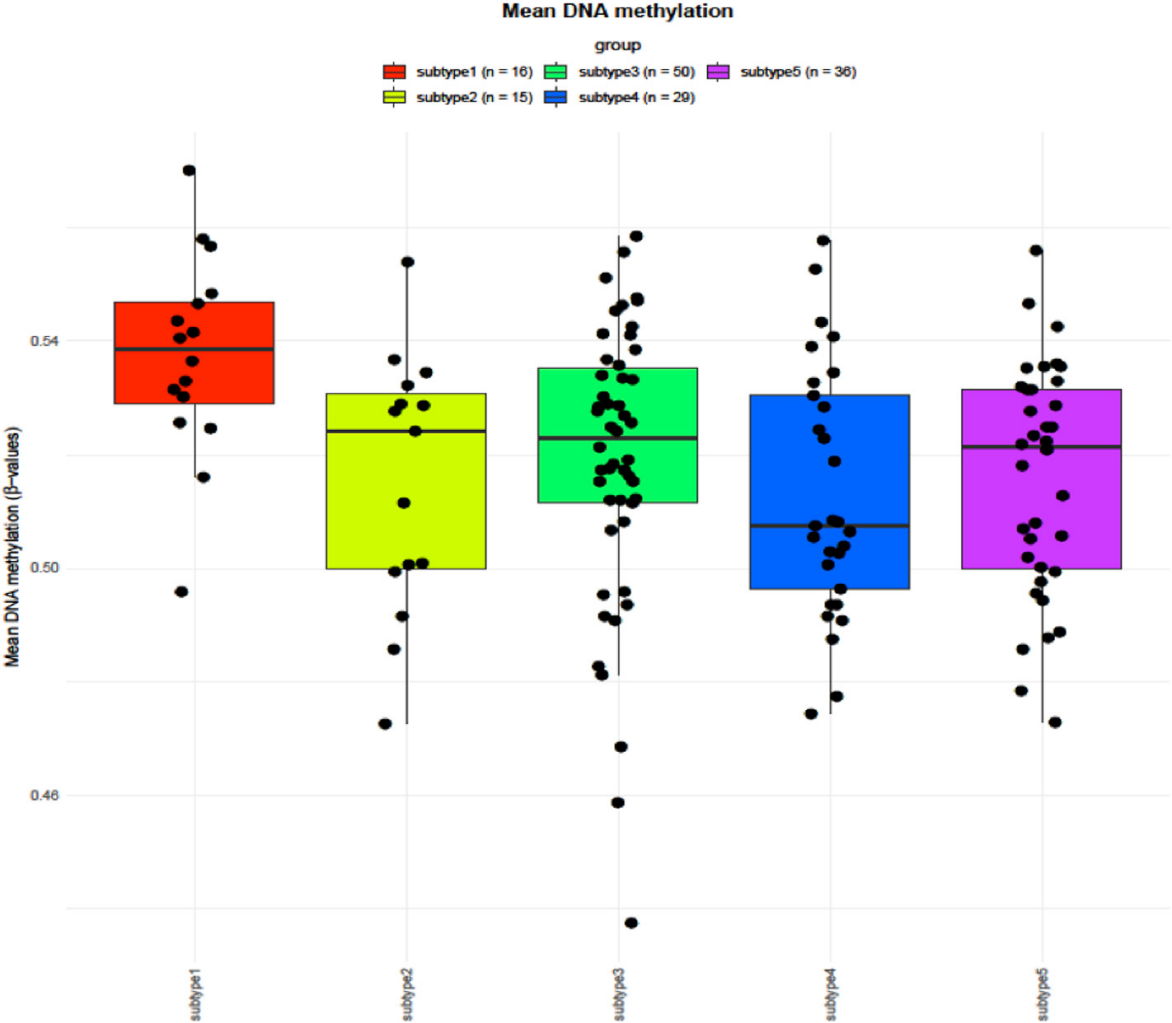
Visualizing differences in mean methylation pattern between comparison groups obtained by subtyping using TCGAbiolink package. We can see from the images that means methylation value between the obtained subtypes shows differences. Thus showing us the significance of obtained subtypes I terms of methylation.

**Figure 7:**
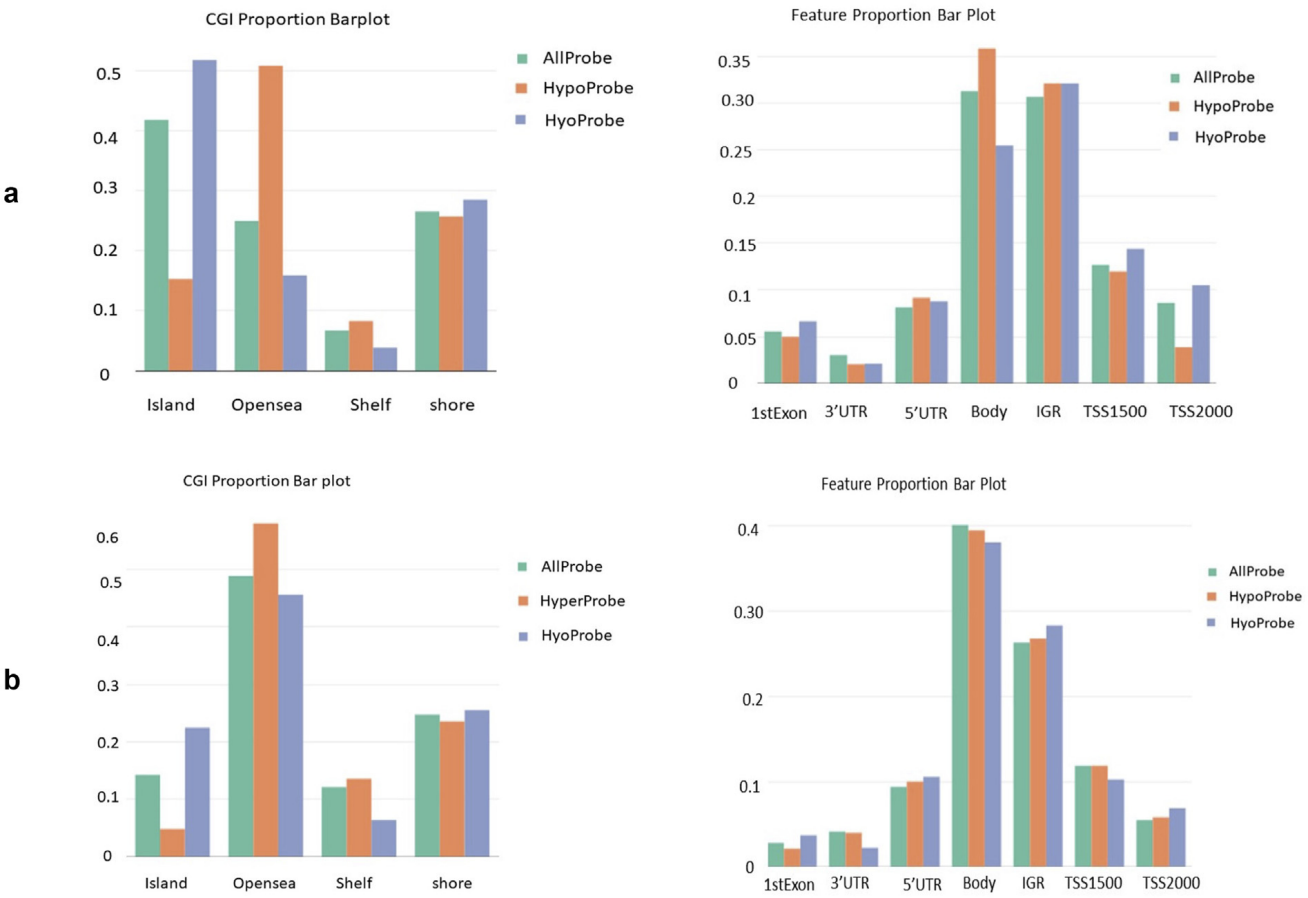
Differential methylation analysis between subtypes obtained by clustering using ChAMP package shows differences in methylation pattern in different regions of the genome for: a) Subtype I. b.) Subtype II.

TCGAvisualize_meanMethylation function of TCGAbiolinks was used for visualizing differences in the mean methylation value of patients between comparison groups (Figure 6). It shows differences in the overall methylation expression between the subtypes obtained from the clustering analysis. The distribution of DMRs (hyper-methylation and hypo-methylation) was obtained using the ChAMP. The landscape of methylation include methylation in the promoter region, Transcription Start Site (TSS), intergenic, island, shell, and shore region. In the subtype I, there are 39128 DMPs out of which hypo-methylation in CpG island is higher as compared to that of hyper-methylation. In open sea, hypermethylation is higher as compared to hypomethylation. The shelf region is characterized by low methylation, while in the shore region, there is an equal distribution of hypermethylation and hypomethylation. Exon, 3′UTR, 5′UTR, Intergenic region, and TSS1500 have equal hypomethylation and hypermethylation. Gene body and TSS2000 have more hypermethylation as compared to hypomethylation (Figure 7a). In subtype II, there are 11011 DMPs, out of which there is more hypo-methylation in CpG island as compared to that in the case of hyper-methylation. In the open sea, hypermethylation is higher as compared to hypomethylation. In the shell region, there is low methylation and in the shore region, there is an equal distribution of hyper as well as hypomethylation. The exon and 5′UTR have a low methylation density, where the distribution of hyper and hypomethylation is almost equal. 3′UTR has more hypermethylation as compared to hypomethylation. The Gene body has a high density and equal distribution of hyper and hypomethylation. The intergenic region has a high density and hypermethylation is more as compared to hypomethylation. Both TSS1500 and TSS2000 have more hypermethylation as compared to hypomethylation (Figure 7b). In the subtype III, there are 7721 DMPs, out of which there is more hypomethylation than hypermethylation in CpG islands. In the open sea, hypermethylation is more as compared to hypomethylation. In the shelf region, hypermethylation is more as compared to hypomethylation and in the shore region, hypermethylation, as well as hypomethylation, are equally abundant. In the subtype III, exon region, 5′UTR, TSS1500 and TSS2000 have more hypomethylation as compared to hypermethylation. 3′UTR gene body and intergenic region have more hypermethylation as compared to hypomethylation (supplementary file I-Figure S4). In subtype IV, there are 6731 DMPs, out of which there is more hypermethylation than hypomethylation in the CpG island region. In the open sea region, hypo-methylation is greater than hyper-methylation whereas, in the shelf and shore region, hypo-methylation is more abundant as compared to hyper-methylation. In subtype IV, the exon, 3′UTR, 5′UTR, gene body, intergenic region, and TSS1500 have low hypermethylation probes as compared to high hypermethylation probes whereas only TSS2000 has more hypermethylation as compared to hypomethylation. In subtype V, there are 6728 DMPs, out of which there is more hypermethylation as compared to hypomethylation in CpG. In the open sea region, hyper-methylation is more as compared to hypo-methylation. In the shell region, hypo-methylation is more as compared to hyper-methylation while in the shore region, distribution is equal (supplementary file I-Figure S5). In the subtype V, the exon, 5′UTR, TSS1500, and TSS2000 have more hypermethylation as compared to hypomethylation, whereas 3′UTR, gene body, and intergenic region have lower hypermethylation sites as compared to hypomethylation (supplementary file I-Figure S6). Overall hypo-methylation expression landscape between all subtypes shows that subtype III has most hypomethylation in island region whereas subtype II and subtype III have in open sea region (Supplementary file I, S7). Overall hyper-methylation expression landscape between all subtypes shows that subtype II has hyper methylation open sea region whereas subtype IV and subtype V in island region (Supplementary file I, S8). There is no difference in hypomethylation expression in shore region. Thus, there are difference in methylation landscape in different subtypes obtained by our analysis.

### 3.6 Correlation analysis

Correlation analysis was performed between DNA methylation and gene expression to determine the extent to which DNA methylation influenced gene expression in the subtypes obtained in our analysis. Differentially methylated CpGs and the differentially methylated genes were used for correlation analysis for each of the obtained subtypes using starburst function. Correlation is observed for each of the subtype (I, II, III, IV, V) individually, based on its DMR and DEG profile. Therefor obtained gene signatures and pathway regulated by them has been used to characterize that particular subtypes. The starburst performs a correlation analysis of DMR and DEG to find out the genes that have a significant correlation with the expression pattern of DMR. In the subtype I there are total 768 gene signature that have a significant correlation with methylation expression pattern, out of which 36 genes show hypermethylation and 732 genes show hypomethylation (supplementary file I-Figure S9, supplementary file II-subtype I). There is a gene signature with 254 genes in subtype II that has a significant correlation with methylation expression pattern, out of which 204 genes show hypermethylation and 50 genes show hypomethylation (supplementary file I-Figure S10, supplementary file II-subtype II). In subtype III, there is gene signature with 76 genes with significant correlation with methylation expression pattern, out of which 26 genes show hypermethylation and another set of 50 genes show hypomethylation (supplementary file I-Figure S11, supplementary file II-subtype III).

There is a gene signature with 390 genes in Subtype IV with a significant correlation with methylation expression pattern, out of which 364 genes show hypermethylation and 26 genes show hypomethylation (supplementary file I-Figure S12, supplementary file II-subtype IV). In subtype V there are total 148 gene signature that have significant correlation with methylation expression pattern, out of which 122 genes show hypermethylation and 26 genes show hypomethylation (supplementary file I-Figure S13, supplementary file II-subtype V). Our DMP and correlation analysis show there is direct relation between proportion of differentially methylated probe to gene signatures obtained by correlation analysis for individual subtypes (supplementary file I, S14).

### 3.7 Functional annotation of PDAC subtypes using clusterprofiler

Correlation analysis of the obtained subtypes resulted in a distinct pattern of gene expression and methylation. Our analysis results in subtypes with considerable overlap and correlation with the previously reported pancreatic ductal adenocarcinoma subtypes with their characteristic pathways. A novel gene signature was reported for each of the obtained subtypes. This analysis has tried to characterize the obtained subtypes based on the available literature regarding the gene signatures.

### 3.8 Subtype I – ADEX subtype genes

**Figure 8:**
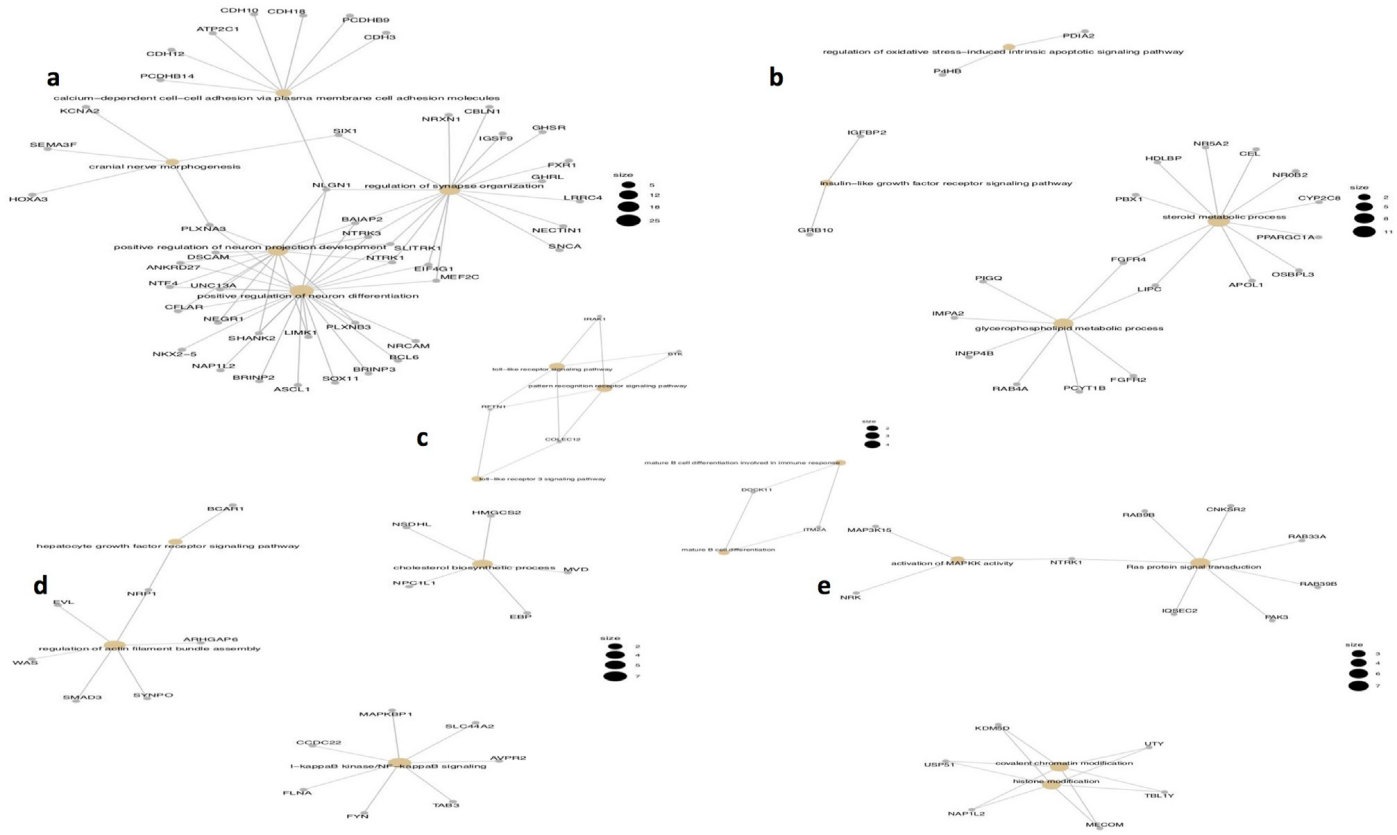
a) Gene ontology analysis of the gene set obtained from correlation analysis using clusterprofiler package shows subtype I having similarity to major pathways of ADEX subtype. b) Gene ontology analysis of the gene set obtained from correlation analysis using clusterprofiler package shows subtypes II having similarity to the major pathways of classical/pancreatic progenitor subtype. c) Gene ontology analysis of the gene set obtained from correlation analysis using clusterprofiler package show subtypes III having similarity to the major pathways of Immunogenic subtype. d) Gene ontology analysis of the gene set obtained from correlation analysis using clusterprofiler package shows subtypes IV having similarity to the major pathways of stroma subtype. e) Gene ontology analysis of the gene set obtained from correlation analysis using clusterprofiler package for shows subtypes V having similarity to the major pathways of squamous subtype.

Pancreatic progenitor displays a transcriptional network of early pancreatic development (FOXA2/3 and PDX1) [13]. Subtype I display upregulation of genes involved in the latter stages of pancreatic development, differentiation and endocrine differentiation (NEUROG1 and NKX2-2) similar to ADEX subtypes. ADEX subtype includes genes responsible for endocrine/exocrine differentiation of pancreas [13]. The key genes identified in subtype I include HOXA3. The HOXA3 family genes are involved in pancreas development and upregulated in pancreatic cancer. The human protein atlas also shows the HOX gene family's oncogenic role with reduced survival (Figure 8a) [51].

Another signature gene in the subtype is CDH3, a classic cadherin protein, a member of a single-span transmembrane domain glycoprotein, involved in cell-cell adhesion. It is hypomethylated in the promotor region (Figure 8a) [52].

LIMK1 is a serine/threonine kinase that regulates actin filament dynamics. It phosphorylates and deactivates de-polymerization factors such as CFL1 and CFL2 resulting in the stabilization of actin filament (Figure 8a) [53]. It is involved in metastasis and tumor-cell induced angiogenesis in pancreatic cancer [54].

SLC17A7, SLC25A5, SLC35A2 are series of transporters expressed in organ and tissue of the digestive tract involved in the uptake of a small molecule (Figure 8a) [55, 56, 57].

NRP1 is a prognostic marker in stomach cancer, cervical cancer, renal cancer and glioma (Figure 8a) [58]. NRP1 is a prognostic marker, hypomethylated and co-expressed with PDGFRB resulting in reduced gastric cancer survival [59].

### 3.9 Subtype II genes

Subtype II is similar to the classical/pancreatic progenitor subtype. The classical/progenitor subtype includes transcription factors that determine the pancreas endoderm fate [13]. The key genes identified in subtype II are: FGFR2, which acts as a cell surface receptor for fibroblast growth factors regulating cell differentiation, proliferation, migration and embryonic development (Figure 8b) [60]. It is responsible for activating MAPK and AKT1 signaling pathway by phosphorylation of FRS2 that activates RAS, MAPK and ERK [60].

Other important genes in the subtype include PDIA2, a member of endoplasmic reticulum family disulphide isomerase that catalyzes protein folding by thiol-disulphide interaction changes specific to the pancreas (Figure 8b) [61]. It is involved in various tumors and is specific to the pancreas. PDIA2 is engaged in multiple tumors, according to recent research [62]. Subtype II is associated with the expression of genes related to digestive enzymes, characteristic of the exocrine pancreatic function such as CYPA1, CYPB.

### 3.10 Subtype III genes

Subtype III is similar to the immunogenic subtype. The subtype is enriched in key immunological genes. The key subtype III genes are:

BTK encodes Bruton's Tyrosine Kinase that regulates cytokine signaling by PLCG phosphorylation in close cooperation with B cell linker protein BLNK resulting in B lymphocyte development, differentiation and signaling (Figure 8c) [63]. The therapeutic role of BTK inhibition has been reported in PDAC [64]. Mice model studies have shown the therapeutic role of BTK inhibition in PDAC [63].

Another key gene in the signature includes IRAK1, a serine-threonine kinase involved in toll-like receptor and IL-1R signaling that initiates innate immune response against foreign pathogens. TLR activation helps in recruiting MYD88 that phosphorylates IRAK1, which brings together IRAK4, MYD88 and tollip and leads to NK-KB activation (Figure 8c) [65].

DOCK 11 is a guanine nucleotide exchange factor and is important for B cell development and plays a role in the development of B cell in the marginal zone (Figure 8c) [66, 67]. Dock180 contributes to ovarian carcinogenesis and since its overexpression is correlated with poor patient survival, it can be a potential prognostic marker and therapeutic target [68]. High DOCK2 expression is involved in better prognosis in AML [69, 70].

### 3.11 Subtype IV – Stroma (Microenvironment of tumor) genes

One of the important features of PDAC is non-tumor cells collectively known as stroma responsible for its progression. This feature contributes to the heterogeneity associated with PDAC resulting in less established patients. Therefore, it is important to recognize the key molecular features and biological processes responsible for the heterogeneity and PDAC progression [71]. The key genes identified in subtype IV are:

TAB3 gene forms a ternary complex with protein kinase MAPK3K7/TAK1 leading to the stimulation of pro-inflammatory cytokine and NF-kappa signaling activation (Figure 8d). TAB2 gene exhibits polymorphism and is associated with ovarian cancer susceptibility [72]. TAB3 gene overexpression is associated with poor survival in human esophageal squamous cell carcinoma [73].

SMAD3 is a potential biomarker in PDAC, which promotes cancer's malignant potential through EMT induction in malignant cells [74]. Hepatocyte growth factor promotes pancreatic cancer's growth and behavior by promoting the ductal phenotype (Figure 8d) [75].

Another important gene in the subtype, NSDHL, encodes a gene localized in the endoplasmic reticulum involved in cholesterol biosynthesis (Figure 8d) [76].

### 3.12 Subtype V genes

Subtype V shows resemblance to the squamous subtype enriched in major pathways like MAPK, Ras protein signaling and chromatin modification. The key genes identified in subtype V are:

MAP3K15 gene is a member of the mitogen-activated protein kinase that is involved in the protein kinase signal transduction pathway (Figure 8e). MKK3/6-p38 MAPK-caspase signaling pathway activation results in the induction of apoptosis induced by Gemcitabine in human pancreatic cancer, serving as a novel marker [77].

PAK3 is a serine-threonine protein kinase that regulates various signaling pathways such as cell migration, cytoskeleton regulation and cell cycle regulation (Figure 8e). PAK3 acts on Ser473-Akt kinase regulating the Akt-GSK3β-β-catenin signaling in several pancreatic cancer cell lines [78].

KDM5D gene is a histone demethylase that plays a major role in histone modification by demethylation of lysine of histone H3 (Figure 8e) [79]. It is found to promote pancreatic cancer by modification of the epigenetic landscape [80].

### 3.13 Survival curve of subtypes

Survival analysis of the five subtypes was obtained using Kaplan-Meier analysis. Subtype V has the worst clinical outcome as compared to the other subtypes in the survival analysis. No significant differences were observed for survival analysis between samples classified into subtype III and subtype IV (supplementary file II-Figures S17-S18). Subtype II has the best clinical outcomes compared to the other subtypes (supplementary file II-Figure S16). Subtype I has the worst clinical outcomes than that of subtype II, III, and IV. Subtype I show a drastic decline in survival around 1000 days (supplementary file II-Figure S15).

## 4 Discussion

Pancreatic cancer treatment is faced with the significant challenge of heterogeneity in the genomic profile of patients. However, the advancement of molecular profiling techniques has led to a better understanding of heterogeneity in pancreatic cancer.

Compared to the traditional classification technique based on staining and histochemical studies, these methods classify samples into distinct subgroups based on molecular characteristics having clinical implications. However, heterogeneous classification results may be obtained by varying patient cohorts, gene expression platforms, and clustering methods. Different methodologies and platforms have resulted in different classifications of PDAC and it has been classified into two to six subtypes by other groups. But they have their limitations and inconsistency. This prompts a better classification of PDAC, where the role of data integration comes into play. Data integration helps in identifying latent factors hidden across different levels of data and thus helps in better identification of the heterogeneity in data. PDAC has a characteristic feature of abundant stroma that constitutes a major percentage of the tumor mass. The presence of microenvironment, stroma and tumor cell infiltrate make PDAC highly heterogeneous. Besides, PDAC also has infiltrative natures having normal pancreatic components along with the tumor. Tumor microenvironment cells molecular profiling may help define molecular subgroups and identify carcinogenic mechanisms based on mRNA expression and their epigenetic regulation. Studies involving various other datasets involving gene expression, DNA methylation, miRNA and long non-coding RNA have shown putative markers important for survival in PDAC. Genome-wide methylation studies have been performed in TCGA pancreatic cancer datasets involving all pancreatic cancer dataset and normal samples providing three cluster solution with significant insights showing stage-specific subtyping like histologic grade G1 and T3 stage subtype and have shown important gene and methylation on histone modifying core genes like histone reader, editor and eraser genes [81]. In comparison, we have removed the heterogeneity in the TCGA_PAAD data set by removing the non-PDAC samples from PDAC samples. All studies were carried out on matched samples of PDAC. We have applied intNMF to perform an integrative study of varied datasets to perform unsupervised classification of PDAC, which involves two levels of data, namely RNA-Seq and DNA methylation data. We have used all state-of-the-art normalization processes for the DNA methylation data namely SWAN, BMIQ, Noob, and Functional normalization for normalization of DNA methylation data. Amongst these four methods, we showed that SWAN performed best and then we performed integratomics and further downstream studies, which included clustering using intNMF, DEG, DMR, and the correlation study. We propose five molecular and clinical distinct PDAC subtypes and studied survival analysis of these subtypes, based on the integrative clustering approach. Our study improves our understanding of PDAC heterogeneity and further helps decipher the molecular and clinical significance of different subtypes.

The five subtypes emerging out of this analysis correlate properly with the already identified subtypes of PDAC based on Bailey's and Moffitt's classification. This study has shown the methylation landscape in different subtypes of PDAC obtained after clustering and correlation studies.

This study will strengthen our understanding of the impact of methylation landscape of hyper-methylation and hypo-methylation on different gene regions in gene expression profiles of obtained PDAC subtypes. Our study focuses on the role of DNA methylation on gene expression at different loci in the different genes in the heterogeneous PDAC landscape. It will help improve and help in a better understanding of epigenetic regulation on the gene expression in PDAC, using unsupervised classification that will lead to better subtyping, prognosis and personalized medical treatment.

## 5 Conclusion

Our integrative study proposes five biological subtypes of PDAC, with their distinct molecular features and clinical outcomes. The proposed study will lead to better identification of PDAC and help in a better prognosis, personalized treatment and help in delineating the heterogenetic landscape of PDAC. The obtained subtype-specific genes by our analysis have the potential to drive personalized therapies and risk prediction for PDAC patients [82].

## Declarations

### Author contribution statement

Shikha Roy: Conceived and designed the experiments; Performed the experiments; Analyzed and interpreted the data; Contributed reagents, materials, analysis tools or data; Wrote the paper.

Amar Pratap Singh: Conceived and designed the experiments; Analyzed and interpreted the data; Wrote the paper.

Dinesh Gupta: Conceived and designed the experiments; Analyzed and interpreted the data; Contributed reagents, materials, analysis tools or data; Wrote the paper.

### Funding statement

D. Gupta was supported by the Bioinformatics Infrastructure Facility (BIF) at ICGEB, by The Department of Biotechnology, Ministry of Science and Technology (BT/BI/25/066/2012). S. Roy was supported by the 10.13039/501100001332Council for Scientific and Industrial Research, India (IN (09/0512(0212)/2016/EMR-1). A. P. Singh was supported by the Council of Scientific and Industrial Research, India (IN) (09/0512(0209)/2016/EMR-1).

### Data availability statement

Data associated with this study is available online in the public domain in the TCGA resource.

### Declaration of interests statement

The authors declare no conflict of interest.

### Additional information

No additional information is available for this paper.

## Appendix A Supplementary data

supplementary I.docx Supplementary file I: Figure S1: Normalization for Infinium I and Infinium II probe normalizations using BMIQ, Figure S2: Normalization for Infinium I and Infinium II probe normalizations using NOOB, Figure S3: PCA analysis of our integrative datasets showing that principle component does not significant variability in the datasets, Figures S4–S6 showing differential methylation analysis of individual subtypes in different region of genome, Figure S7: Comparison of hypo-methylation expression profile of probes between all the subtypes, Figure S8: Comparison of hyper-methylation expression profile of probes between all the subtypes, Figures S9–S13: Correlation plots between methylation and gene expression, Figure S14: Comparison between differentially methylated probes and correlation gene signature for obtained subtypes, Figures S15–S19: Kaplan-Meier survival analysis of the obtained subtypes. supplementary I.docx

supplementary file II.xlsx Supplementary file II: List of significant gene signatures obtained by correlation analysis of each subtype. supplementary file II.xlsx
