# Supplementary material for: Unsupervised subtyping and methylation landscape of pancreatic ductal adenocarcinoma
Source: Heliyon. 2021 Jan 18;7(1):e06000. doi: 10.1016/j.heliyon.2021.e06000 (PMC7820567; doi:10.1016/j.heliyon.2021.e06000)
Supplement: supplementary I.docx — Supplementary file I: Figure S1: Normalization for Infinium I and Infinium II probe normalizations using BMIQ, Figure S2: Normalization for Infinium I and Infinium II probe normalizations using NOOB, Figure S3: PCA analysis of our integrative datasets showing that principle component does not significant variability in the datasets, Figures S4–S6 showing differential methylation analysis of individual subtypes in different region of genome, Figure S7: Comparison of hypo-methylation expression profile of probes between all the subtypes, Figure S8: Comparison of hyper-methylation expression profile of probes between all the subtypes, Figures S9–S13: Correlation plots between methylation and gene expression, Figure S14: Comparison between differentially methylated probes and correlation gene signature for obtained subtypes, Figures S15–S19: Kaplan-Meier survival analysis of the obtained subtypes. [file mmc1.docx]

***Supplementary File I***


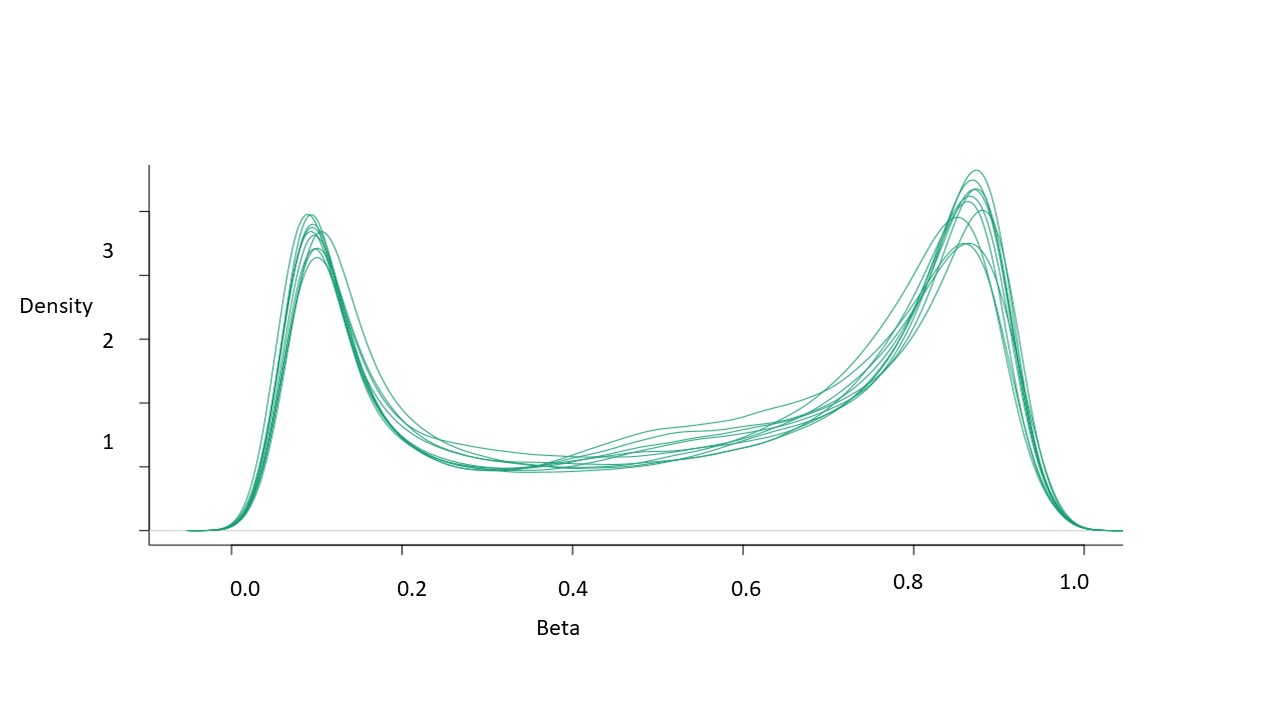


Figure S1: Normalization for Infinium I and Infinium II probe normalizations using BMIQ.


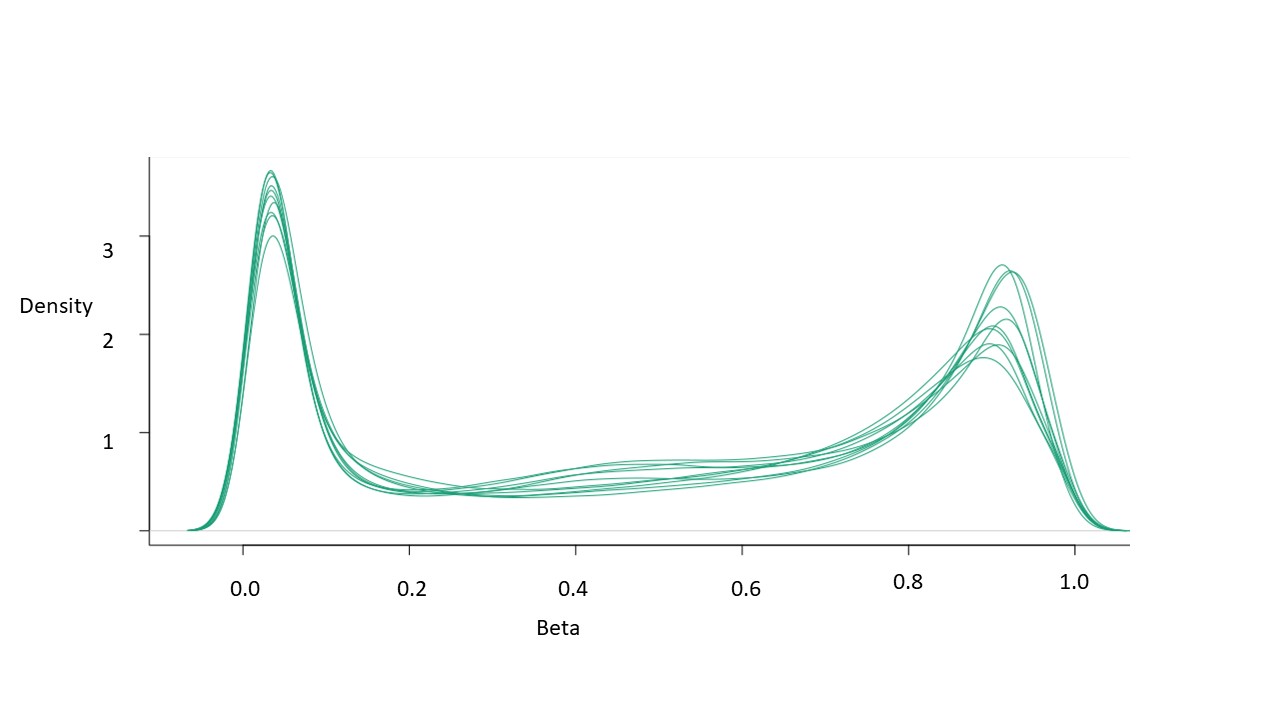


Figure S2: Normalization for Infinium I and Infinium II probe normalizations using NOOB.


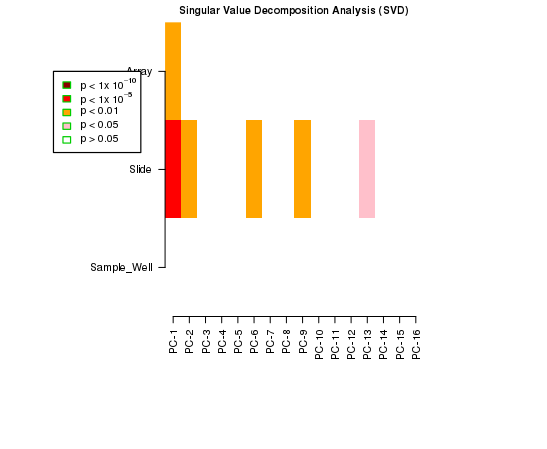


Figure S3: PCA analysis of our integrative datasets showing that principle component does not significant variability in the datasets.


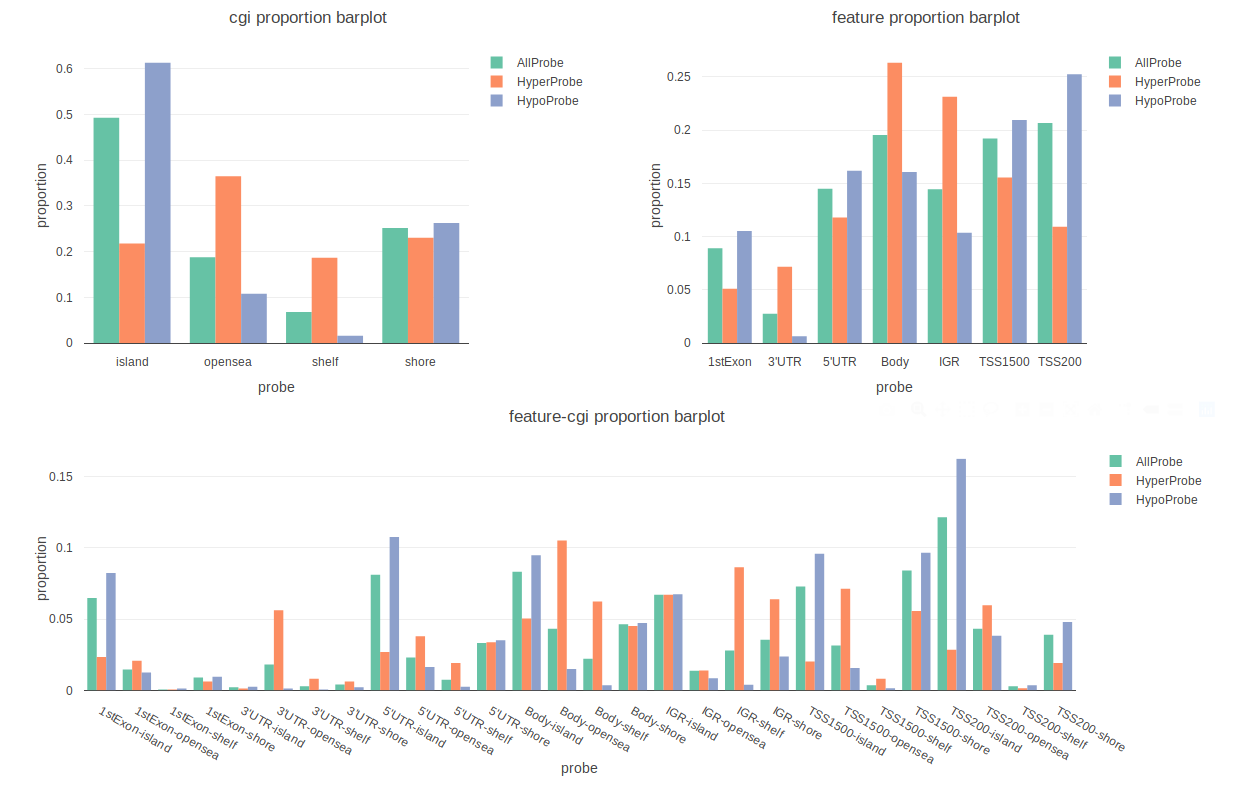


Figure S4: Differential methylation analysis between subtypes obtained by clustering using ChAMP package shows differences in methylation pattern in different regions of the genome for subtype III.


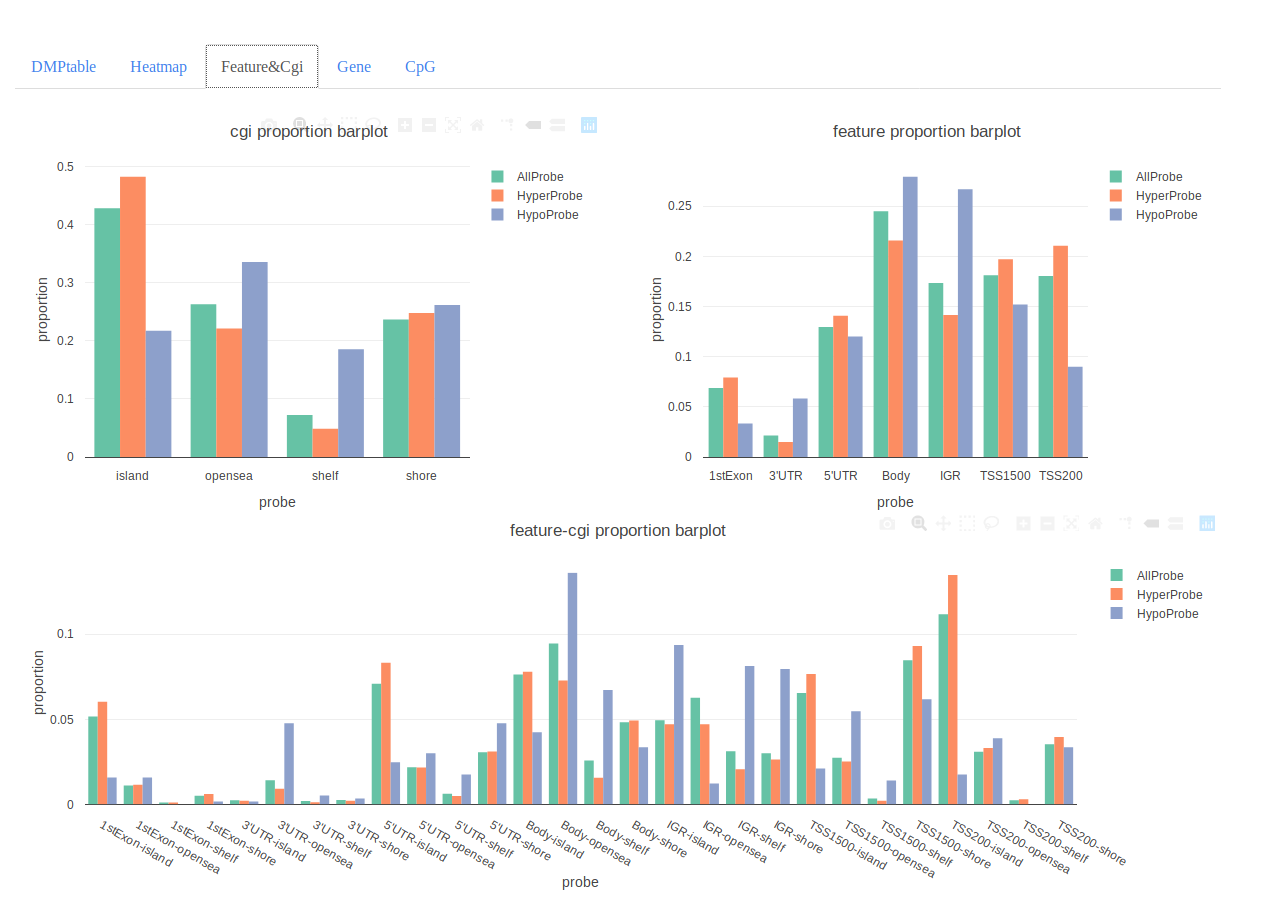


Figure S5: Differential methylation analysis between subtypes obtained by clustering using ChAMP package shows differences in methylation pattern in different regions of the genome for subtype IV.


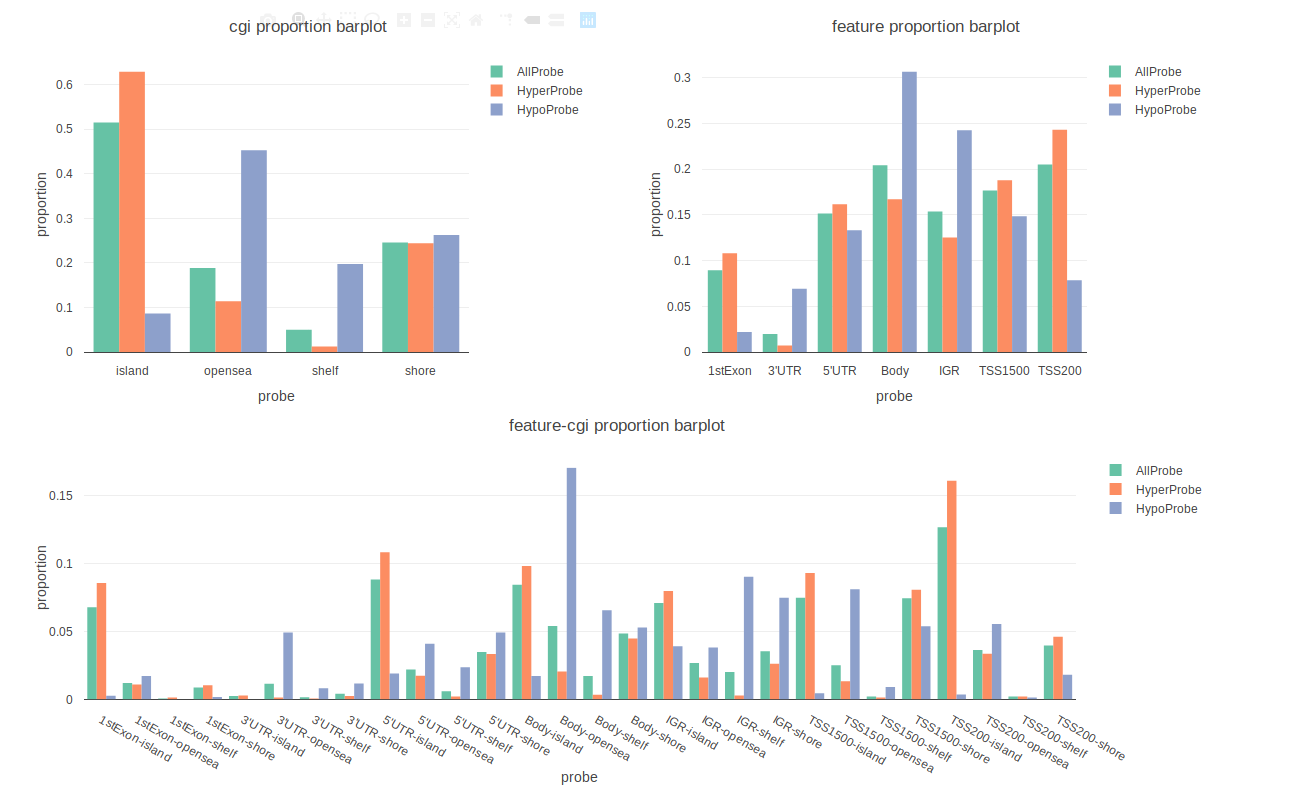


Figure S6: Differential methylation analysis between subtypes obtained by clustering using ChAMP package shows differences in methylation pattern in different regions of the genome for subtype V.

Figure S7: Comparison of hypo-methylation expression profile of probes between all the subtypes.

Figure S8: Comparison of hyper-methylation expression profile of probes between all the subtypes.

Figure S9: In Subtype I there was total 768 genes in the gene signature that had significant correlation with methylation expression pattern, out of which, 42 genes have hyper methylation and 716 genes hypo methylation.

Figure S10: In Subtype II there was total 254 gene signature that had significant correlation with methylation expression pattern, Out of which 204 gene signature having hyper methylation and 50 gene signature was having hypo methylation.

Figure S11: In Subtype III there was total 76 gene signature that had significant correlation with methylation expression pattern, out of which 26 gene signature having hyper methylation and 50 gene signature was having hypo methylation

Figure S12: In Subtype IV there was total 390 gene signature that had significant correlation with methylation expression pattern, Out of which 360 gene signature having hyper methylation and 28 gene signature was having hypo methylation.

Figure S13: In Subtype V there was total 148 gene signature that had significant correlation with methylation expression pattern, out of which 122 gene signature having hyper methylation and 26 gene signature was having hypo methylation.

Figure S14: Comparison between differentially methylated probes and correlation gene signature for obtained subtypes.

Figure S15: Survival analysis of Subtype I as compared to all other subtypes, obtained using Kaplan-Meier analysis.

Figure S16: Survival analysis of Subtype II as compared to all other subtypes, obtained using Kaplan-Meier analysis.

Figure S17: Survival analysis of Subtype III as compared to all other subtypes, obtained using Kaplan-Meier analysis.

Figure S18: Survival analysis of Subtype IV as compared to all other subtypes, obtained using Kaplan-Meier analysis.

Figure S19: Survival analysis of Subtype V as compared to all other subtypes, obtained using Kaplan-Meier analysis.
